# Supplementary material for: A Prediction Model for Preoperative Risk Assessment in Endometrial Cancer Utilizing Clinical and Molecular Variables
Source: Int J Mol Sci. 2019 Mar 9;20(5):1205. doi: 10.3390/ijms20051205 (PMC6429416; doi:10.3390/ijms20051205)
Supplement: Supplementary file 1 [file ijms-20-01205-s001.docx]

**Appendix C**

Example of the R code used to do the variable selection, lasso prediction analysis and replication in TCGA, and prediction validation in TCGA. For this example we used only clinical and miRNA normalized expression data. Other data can be added to the analysis as long is ordered by the same sample/patient ID (rows). To use this code copy the following lines into you preferred code editor.

#############################################################################################

#############################################################################################

#############################################################################################

###### #############

###### For the analysis we will use a example integrating clinical #############

###### variables and miRNA expression. The analysis will be divided in: #############

###### 1. Variables selection with caret package; #############

###### 2. Prediction model analysis integrating UI clinical data and #############

###### miRNA data #############

###### 3. Replication of the analysis in TCGA data (clinical and miRNA) #############

###### 4. Validation of UI model into TCGA data (clinical and miRNA) #############

###### #############

#############################################################################################

#############################################################################################

#############################################################################################

#############################################################################################

###### 1. Variable selection with caret package; #############

#############################################################################################

#Go to the directory

setwd("C:/Directory where you file with the data is")

## The data file shold contain a first column with the label (low vs high risk).

## Then, the next columns will contain normalized miRNA expressions.

## Rows should have all patients/samples

library(caret)

set.seed(1234)

data <- read.table("endo_miRNA.txt", row.names=1, header=T, sep = "\t", na.strings="")

################ Univariate variable selection with CARET ###################################

y <- as.factor(data[,1])

x <- data.matrix(data[,2:dim(data)[2]])

ctrl <- sbfControl(functions=caretSBF, method='repeatedcv', number=10, repeats=10,

allowParallel=TRUE, multivariate=FALSE)

fit<-sbf(x, y, sbfControl = ctrl)

## We could print the results so we understand whcih variables have been selected.

## Also we will have some information about how the seelction was performed

sink('results_miRNA.txt')

fit

fit$fit

fit$optVariables

sink()

#############################################################################################

###### 2. Prediction model analysis integrating UI clinical data and #############

###### miRNA data #############

#############################################################################################

library(glmnet)

## The data file shold contain a first column with the label (low vs high risk), as before ('Risk')

## We will only include those variables that have been selected by the previous step #1 (CARET)

## Rows should have all patients/samples

######################## Clinical ###########################################################

z <- read.table("clin_select.txt", header=T, row.names=1, sep = "\t", na.strings=c("","n/a"))

z$Risk <- relevel(z$Risk, ref = "LR")

clin <- z

######################## miRNA ###############################################################

mirna <- read.table("miRNA_select.txt", header=T, row.names=1, sep = "\t", na.strings=c("","n/a"))

mir <- mirna[, 2:dim(mirna)[2]]

z <- mir[intersect(row.names(mir),row.names(clin)),]

w <- clin[intersect(row.names(mir),row.names(clin)),]

cx <- cbind(w,z)

cc <-na.omit(cx)

y <- cc[,'Risk']

var <- data.matrix(cc[,2:dim(cc)[2]])

clin_la <- cv.glmnet(var,y,family="binomial", type.measure="auc", nfolds=6)

plot(clin_la, s=clin_la$lambda.min, cex.axis=1.5)

coef(clin_la, s=clin_la$lambda.min)

clin_la

#############################################################################################

###### 3. Replication of the analysis in TCGA data (clinical and miRNA) #############

#############################################################################################

## The data file shold contain a first column with the label (low vs high risk), as before ('Risk')

## The data file shold contain TCGA data for replication with the same variables included in

## the previous step #2 (same variabled than in UI)

## Rows should have all patients/samples

####################### Clinical ############################################################

z <- read.table("tcga_clin_select.txt", header=T, row.names=1, sep = "\t", na.strings=c("","n/a"))

z$Risk <- relevel(z$Risk, ref = "LR")

clin <- z

####################### miRNA ###############################################################

mirna <- read.table("tcga_miRNA_select.txt", header=T, row.names=1, sep = "\t", na.strings=c("","n/a"))

mir <- mirna[, 2:dim(mirna)[2]]

z <- mir[intersect(row.names(mir),row.names(clin)),]

w <- clin[intersect(row.names(mir),row.names(clin)),]

cx <- cbind(w,z)

cc <-na.omit(cx)

y <- cc[,'Risk']

var <- data.matrix(cc[,2:dim(cc)[2]])

clin_la <- cv.glmnet(var,y,family="binomial", type.measure="auc", nfolds=10)

plot(clin_la, s=clin_la$lambda.min, cex.axis=1.5)

coef(clin_la, s=clin_la$lambda.min)

clin_la

#############################################################################################

###### 4. Validation of UI model into TCGA data (clinical and miRNA) #############

#############################################################################################

library(glmnet)

#############################################################################################

####### First build the model in UI with Clinical and miRNA data: ci.mi ####################

#############################################################################################

## The data file shold contain a first column with the label (low vs high risk), as before ('Risk')

## We will only include those variables that have been selected by the previous step #1 (CARET)

## Rows should have all patients/samples

######################## Clinical ###########################################################

z <- read.table("clin_select.txt", header=T, row.names=1, sep = "\t", na.strings=c("","n/a"))

z$Risk <- relevel(z$Risk, ref = "LR")

clin <- z

######################## miRNA ###############################################################

mirna <- read.table("miRNA_select.txt", header=T, row.names=1, sep = "\t", na.strings=c("","n/a"))

mir <- mirna[, 2:dim(mirna)[2]]

z <- mir[intersect(row.names(mir),row.names(clin)),]

w <- clin[intersect(row.names(mir),row.names(clin)),]

cx <- cbind(w,z)

cc <-na.omit(cx)

y <- cc[,'Risk']

var <- data.matrix(cc[,2:dim(cc)[2]])

c.mi <- cv.glmnet(var,y,family="binomial", type.measure="auc", nfolds=6)

##############################################################################################

###### Then validate the model built in UI (ci.mi) with TCGA Clinical and miRNA data #########

##############################################################################################

library(pROC)

## The data file shold contain a first column with the label (low vs high risk), as before ('Risk')

## The data file shold contain TCGA data for replication with the same variables included in

## the previous step when we bult the model in UI (same variabled than in UI)

## Rows should have all patients/samples

####################### Clinical #############################################################

z <- read.table("tcga_clin_select.txt", header=T, row.names=1, sep = "\t", na.strings=c("","n/a"))

z$Risk <- relevel(z$Risk, ref = "LR")

clin <- z

####################### miRNA ################################################################

mirna <- read.table("tcga_miRNA_select.txt", header=T, row.names=1, sep = "\t", na.strings=c("","n/a"))

mir <- mirna[, 2:dim(mirna)[2]]

z <- mir[intersect(row.names(mir),row.names(clin)),]

w <- clin[intersect(row.names(mir),row.names(clin)),]

cx <- cbind(w,z)

cc <-na.omit(cx)

y.val <- cc[,'status']

val <- data.matrix(cc[,2:dim(cc)[2]])

response <- predict.cv.glmnet (c.mi, val, s="lambda.min", type="response")

r.mi <- roc(y.val, response[,1])

r.mi

ci <- ci.auc(y.val, response[,1])

ci.thresholds(r.mi)

plot(r.mi)

ci.coords(r.mi,x=0.9, input='sen', ret=c("spec", "accuracy", "npv", "ppv")
